# Electrical Stimulation Promotes Stem Cell Neural Differentiation in Tissue Engineering

**DOI:** 10.1155/2021/6697574

**Published:** 2021-04-20

**Authors:** Hong Cheng, Yan Huang, Hangqi Yue, Yubo Fan

**Affiliations:** ^1^Beijing Advanced Innovation Center for Biomedical Engineering, Key Laboratory for Biomechanics and Mechanobiology of Chinese Education Ministry, School of Biological Science and Medical Engineering, Beihang University, No. 37, Xueyuan Road, Haidian District, Beijing 100083, China; ^2^School of Engineering Medicine, Beihang University, No. 37, Xueyuan Road, Haidian District, Beijing 100083, China

## Abstract

Nerve injuries and neurodegenerative disorders remain serious challenges, owing to the poor treatment outcomes of *in situ* neural stem cell regeneration. The most promising treatment for such injuries and disorders is stem cell-based therapies, but there remain obstacles in controlling the differentiation of stem cells into fully functional neuronal cells. Various biochemical and physical approaches have been explored to improve stem cell-based neural tissue engineering, among which electrical stimulation has been validated as a promising one both in vitro and in vivo. Here, we summarize the most basic waveforms of electrical stimulation and the conductive materials used for the fabrication of electroactive substrates or scaffolds in neural tissue engineering. Various intensities and patterns of electrical current result in different biological effects, such as enhancing the proliferation, migration, and differentiation of stem cells into neural cells. Moreover, conductive materials can be used in delivering electrical stimulation to manipulate the migration and differentiation of stem cells and the outgrowth of neurites on two- and three-dimensional scaffolds. Finally, we also discuss the possible mechanisms in enhancing stem cell neural differentiation using electrical stimulation. We believe that stem cell-based therapies using biocompatible conductive scaffolds under electrical stimulation and biochemical induction are promising for neural regeneration.

## 1. Introduction

Nerve diseases, including axon loss, nerve injury, and degenerative nerve disease, are a severe economic burden to society. Current medical and surgical strategies and physiotherapy are common treatments for nerve diseases. These strategies alleviate pain after nerve injury, maintain the continuity of nerves, and delay disease progression but are difficult to perform, time-consuming, expensive, and do not always result in sufficient functional recovery and nerve regeneration. Stem cells, including neural stem cells (NSCs) and other exogenous multipotent stem cells, have the ability to differentiate into neural lineages. Accumulating evidence has indicated that stem cell therapy is a promising option in regenerating damaged neurons, assisting functional restoration through the differentiation of stem cells into neurons and glial cells, secreting cytokines and growth factors, activating endogenous repair through immunomodulation, and inhibiting cell apoptosis and fibrosis. In addition, numerous clinical trials have been initiated to evaluate the safety and efficacy of stem cell therapy in patients with various nerve diseases.

A prerequisite in applying stem cells to nerve tissue engineering is controlling the differentiation of stem cells into neural cells with precision and efficacy. Many biophysical strategies, particularly electrical stimulation (ES), have been made to improve the efficiency of stem cell neural differentiation. ES has been demonstrated capable of enhancing the proliferation and differentiation of stem cells, inducing guided cell migration, and promoting the growth and elongation of neurites [1–4]. In addition, low-frequency ES has also been proven effective clinically in regenerating nerves, hence leading to regeneration and functional recovery [5]; however, the effects of ES on stem cell neural differentiation in different studies slightly vary, owing to the fact that ES frequency, duration, voltage, and the conductive and electroactive material applied varied according to the type of stem cells and loading systems. Thus, the optimal setting for the ES of different stem cells for nerve tissue engineering is difficult to specify. In this review, we summarize various methods in delivering ES to achieve stem cell neural differentiation and maturation both *in vitro* and *in vivo*. We also analyse the potential mechanisms of ES in stem cell differentiation. Furthermore, we discuss here our perspectives on the future of the clinical application of ES on stem cells for the treatment of nerve diseases.

## 2. Electrical Stimulation Enhances Stem Cell Neural Differentiation

**Figure 1:**
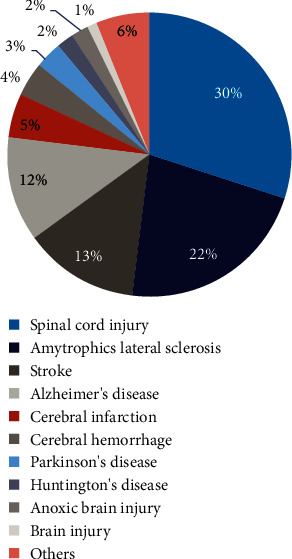
Proportion of different nerve disease types for which MSCs were used as a treatment in clinical trials.

Stem cells can self-renew and differentiate into multiple cell types. In recent decades, many different stem cell types including NSCs, mesenchymal stem cells (MSCs), induced pluripotent stem cells (iPSCs), and embryonic stem cells (ESCs) have been investigated *in vitro* and *in vivo* to assess the therapeutic potential of stem cell therapies [6–10]. Depending on the origin of stem cells, they exhibit different levels of potency. NSCs located in the specific regions of developing and adult human brain are tissue-specific stem cells and can terminally differentiate into all neural lineages, including neurons, astrocytes, and oligodendrocytes [11]. The application of NSCs is considered a promising therapeutic strategy for treating of central nervous system diseases, including Parkinson's disease, Alzheimer's disease, and spinal cord repair [12–14]. Preclinical researches NSCs derived from fetal tissues, ESCs, and iPSCs showed enhanced recovery after stroke [15–17] and comparable neurological disorders [18, 19]. Due to the similarity of iPSCs and human ESCs (hESCs), similar approaches for the induction of their neural differentiation can be used. Hu et al. compared the neural differentiation capacity between iPSCs and hESCs. They found that iPSCs have the same gene expression pattern and period required to differentiate into functional neurons as ESCs but with increased variability and reduced efficiency [20]. Clinical studies in which ESCs and iPSCs were used for the treatment of nerve diseases are listed in Table 1. At present, most clinical research aims to generate iPSCs from patients with nerve disease to establish disease models, and only a few aim to differentiate iPSCs into neurons and glia for cell transplantation. MSCs are the most commonly used stem cells and can be derived from tissues, such as the bone marrow, adipose tissues, and umbilical cord. Some animal studies have shown that transplanted MSCs can migrate to injured sites of the brain, differentiate into neuron-like cells expressing microtubule association protein-2 (MAP2) and glial fibrillary acidic protein (GFAP), and improve neurological function after stroke and spinal cord injury [21, 22]. The differentiation capacity of MSCs from different sources was reportedly not the same. Umbilical cord, bone marrow, and adipose tissue-derived MSCs have been used in clinical research for a number of nerve diseases such as spinal cord injury, amyotrophic lateral sclerosis, and stroke (Figure 1) [23].

There are complex and varied regulatory networks involved in the neural differentiation of stem cells under different conditions. Certainly, the use of growth factors and small molecules remains the predominant method for stem cell differentiation; however, the use of nonbiochemical methods to assist stem cell differentiation has attracted the attention of many researchers. As neurons are electrically active cells, exogenous ES can provide artificial stimulation that transmits electrical charge directly to the cells. The potential positive effect of exogenous ES on nerve regeneration following injury has been extensively studied. It has been shown that ES can improve neural cell proliferation [24] and the function of neurons and Schwann cells when subjected to a voltage gradient during neural development and postinjury [25]. Exogenous ES has been reported to enhance stem cell neuronal migration [26], differentiation [27], neurite outgrowth [28], and intracellular Ca^2+^ dynamics *in vitro* [29]. Regarding *in vivo* applications, due to the lack of effective clinical treatments for nerve injuries and neurodegenerative diseases, ES generated from an external power source or from electroactive materials has been explored as a complement and applied in stem cell therapy and tissue engineering since many years ago. Numerous studies on ES therapy have been conducted in animal models and humans and promising results have been reported [30–32]. Exogenous ES in animal models not only guides the migration of stem cells and stem cell-derived neural cells [33–35], but also significantly contributes to stem cell neuron differentiation [36]. In clinical applications, ES therapy as a nonsurgical therapeutic modality is widely adopted by physical therapists and physicians. A variety of ES models have been developed and applied, based on the power sources, including direct current (DC) electric fields, alternating current (AC) electric fields, and pulsed current electric fields. A better understanding of the fundamental principles underlying the ES regulated stem cell neural differentiation would provide clues for developing new strategies for stem cell therapy and devices for nerve tissue engineering.

### 2.1. Effect of Direct Current on Stem Cell Neural Differentiation

DC indicates that the magnitude and direction of the electric charge is consistent, and it can be produced by batteries, fuel cells, and generators with commutators. Different types of stem cells or their differentiated neuron-like cells respond differently to ES (Table 2). Min et al. reported that a small DC can guide the migration of human iPSCs (hiPSCs) and hESCs with different electrotaxis depending on distinct signalling pathways. They reported that DC stimulation less than 30 mV/mm guided the migration of hiPSCs to the anode in both two-dimensional (2D) and three-dimensional (3D) culture conditions and that the migration rate was voltage-dependent [37], whereas 16 mV/mm DC ES guided the migration of NSCs derived from hESCs to the cathode [35]. In addition, the effect of ES on neural differentiation regulation is cell type specific. The sensitivity of MSCs to the changes in electric field strength was reportedly higher than that of NSCs [38]. More studies are necessary to optimize the parameters of DC for each stem cell type because of the cell type-specific sensitivity to ES.

DC stimulation can also guide NSC migration and enhance NSC differentiation and neural maturation. In a DC electric field of 11.5 V/cm, NSCs tended to specifically differentiate into neurons rather than astrocytes or oligodendrocytes [39]. Kobelt et al. [40] reported that a short duration of ES (10 min/day of DC stimulation at 0.53 or 1.83 V/m) for 2 days enhances neurite outgrowth and *β*III-tubulin and neuronal nuclei (NeuN) expression levels and increases the intracellular Ca^2+^ during stimulation. The effect of short time ES on stem cell neural differentiation was also confirmed in human MSCs (hMSCs). Greeshma et al. [41] used polyaniline (PANI) to establish conductivity in polymeric substrates and provided a short time DC electric filed stimulation (100 mV/cm, 10 min every day for 10 days). Intermittent ES reportedly improves neural-like differentiation of hMSCs with elongated filopodia and increased expression of nestin and *β*III-tubulin [41]. Long-time ES can also enhance stem neural differentiation and maturation. Dong et al. treat NSCs with ES for 3 days at 150 mV/mm, resulting in increased achaete-scute homolog (Ascl1) expression that was further proven to regulate phosphatidylinositol 3-kinase/protein kinase B (PI3K/Akt) pathway in NSCs [42]. In addition, hMSCs also showed increased levels of SOX2, nestin, *β*III-tubulin expression, and Ca^2+^ oscillation after nine days of continuous exposure to 8 mV/mm ES for 20 h/day [43]. Taken together, these studies confirm the ES can induce cell orientation and migration, and enhance the differentiation of stem cells into neural cell lineages. Since the time duration of ES and the amplitude of DC varied among studies, it is difficult to directly compare the DC-mediated effects on stem cell differentiation reported in them. Besides, none of these studies investigated the effect of ES on neural gene expression profiles throughout the whole process. ES may have variable impacts at different differentiation stages, which warrants further investigation.

Exogenous DC stimulation has also been reported to exert a positive effect on nerve function recovery *in vivo*. Yamada et al. demonstrated the potential of ESC to differentiate into mature neurons after injection into the injured spinal cords of adult mice [36]. ES could further improve the function recovery, and 7 days of ES (10 Hz, 0.5–1.0 V), which was performed for 4 h/day, may improve the function of the injured spinal cord in rats [44]. Some data showed that DC stimulation can improve motor function after a stroke [45, 46]; in particular, the improvement is greater in chronic stroke patients [47]. The improvements showed a positive relationship with current and charge density when transcranial DC stimulation (tDCS) was applied [48]. Up to 4 mA of tDCS was considered safe and tolerable for stroke patients [49]. In addition, bilateral cerebellar tDCS was also reported to improve balance in patients with Parkinson's disease [50].

### 2.2. Effect of Alternating Current on Stem Cell Neural Differentiation

AC is the flow of charge that changes direction periodically, and its magnitude reverses along with the current. *In vitro* AC systems use capacitively coupled or inductively coupled designs. The applications of AC stimulation in neural differentiation are summarized in Table 3. In contrast to DC, AC may not have effect on NSC migration, alignment with ES [51]. This may be due to the bidirectional electric field provided by AC. However, Matos et al. found that AC stimulation can improve the viability and neural differentiation of NSCs. The best frequency for mouse NSC viability was 1 Hz, and frequency lower than 1 Hz can increase the ratio of neurons to astrocytes [52]. Furthermore, using a frequency higher than 1–50 Hz, 0.001 kV/cm, AC ES delayed neural differentiation of progenitor cells into astrocytes [53]. According to these studies, a wide range of the frequencies of ES can control the differentiation of stem cells into specific sublineages, which depend on the cell types and culture conditions. Apart from *in vitro* AC stimulation, *in vivo* AC devices were also designed. Repetitive transorbital AC stimulation was used to treat mice with optic nerve injury. After treatment, many large neurons survived with moderate dendritic shrinkage [54].

### 2.3. Effect of Pulsed Current on Stem Cell Neural Differentiation

Pulsed current can be pulsed DC or AC, monophasic or biphasic. Monophasic pulsed current is unidirectional whereas biphasic pulsed current refers to two pulses of current in different directions within one pulse duration or bidirectional. Biphasic current is the most versatile waveform for ES owing to the improved duration, amplitude, and frequency of a pulse. It has been indicated that the parameters of electrical stimulation, including frequency, electrical strength, and duration, should be optimized to improve the effect of ES in regulating stem cell neural differentiation.

Pulsed current has shown remarkable effects on stem cell proliferation, neural differentiation, and axonal outgrowth (Table 4). Similar to effect of DC on stem cell viability, pulsed ES can improve NSC survival and prevent growth factor-induced cell apoptosis [55]. In addition, the effect of pulsed ES on stem cell proliferation is cell type specific. Petrella et al. [38] compared the effects of picosecond pulsed electric field on NSCs and MSCs. Pulsed ES has no influence on MSC proliferation but improves NSC proliferation and astrocyte-specific differentiation by upregulating GFAP after 24 h under 40 kV/cm. Chang KA et al. used [56] used indium tin oxide (ITO) glasses to generate a biphasic electrical stimulator chip. They found that biphasic ES (200 *μ*s pulse duration, 100 Hz) increased not only NSC proliferation but also cell differentiation into NeuN, MAP2, and *β*III -tubulin positive neurons. Tandon et al. used a microarray with ITO electrodes to generate monophasic square-wave pulses (5 V, 1 ms duration per 100 ms) and the pulsed ES facilitated mouse retinal progenitor cell differentiation into mature neurons, thereby increasing *β*III-tubulin expression and Ca^2^ influxes [57].

Pulsed ES also exerts an effect on the differentiation of stem cells into subtypes of neural cells other than neurons. Du et al. reported that 20 Hz of 100 *μ*s pulsed ES enhanced human neural crest stem cell differentiation into Schwann cells and promoted nerve regeneration after cell transplantation [58]. Chang et al. reported that pulsed DC electric fields induce cortical NSCs to simultaneously differentiate into neurons, astrocytes, and oligodendrocytes [59]. In contrast, when NSCs growing on poly (L-lactic-co-glycolic acid) (PLGA)/graphene oxide (GO) conductive composite membranes were stimulated with 500 Hz pulsed current for 1 h every day for 3 days, the NSCs showed differentiation tendency towards neurons comparing to astrocytes [60]. Guo et al. reported that MSCs under pulsed ES (300 V, 30 *μ*A, 0.84 Hz) for 21 days differentiated into neurons and astrocyte-like cells [61]. Furthermore, the effect of pulsed ES was also confirmed in vivo. An implanted pulse generator with real-time triggering capabilities restored walking in patients with lower limb paralysis after spinal cord injury [62]. Taken together, these results indicate that pulsed ES play a critical role in stem cell neural differentiation, as it can increase the length and branching of neurites and regulate differentiation into neural subtypes, depending on stem cell type and pulsed ES formats.

## 3. Effect of Electrical Stimulation through Conductive Material on Neural Differentiation

Restoring nerve function is a great challenge in nerve tissue regeneration. Numerous biomaterials and nanocomponents fulfil the need for achieving the functional differentiation of transplanted stem cells in tissue engineering by mimicking the properties of the microenvironment. Here, we summarize and discuss the electroconductive materials used in nerve tissue regeneration (Table 5). Electroconductive materials have been widely investigated in tissue engineering owing to their high electrical conductivity and ability to generate topographical 2D and 3D structures. Devices can be designed with 2D and 3D chambers for in vitro studies.

### 3.1. Effect of Electrical Stimulation through 2D Conductive Material on Stem Cell Neural Differentiation

Owing to the intrinsic electrical properties of neural cells and positive response under ES, there has been a lot of interest in conductive materials for application in neural tissue engineering and regeneration. ES currents can be traditionally delivered through salt bridges submerged inculture media. Many biocompatible materials such as carbon, platinum, gold, titanium, and silver are commonly used as electrodes. To date, metal nanomaterials have been widely used in various tissue engineering studies. A growing number of studies have developed 2D biomaterial substrates or 3D scaffolds using metal deposits in stem cell-based tissue regeneration. Compared to salt bridges with an electrode system, a conductive polymer material provides direct ES through an interface. Yang et al. deposited a thin layer (150–300 nm groove/ridge) of titanium (Ti) onto nanopatterned polyurethane-acrylate substrate surfaces [63]. Their data indicated that nanotopography synergistically upregulated the expression of neural markers (Tuj1, NeuN, MAP2) and improved the electrophysiological properties and functional maturation of neurons differentiated from human NSCs.

With the rapid development of biomaterials, conductive polymer materials, including polypyrrole (PPy) [64, 65], PANI [66], graphene [67], and carbon nanotubes [68–70], have been explored as substrates with acceptable biocompatibility with neural cells. The conductive polymers can locally deliver electrical stimulus to stem cells and even be conjugated with peptides to enhance stem cell proliferation and differentiation. Chuan et al. reported that NSCs planted on a conductive PLGA/GO composite membrane, showed increased proliferation, neuronal differentiation, and neurite elongation [60]. Peptide-coated PPy neural probes implanted in guinea pig brain promoted the neuron attachment [71]. Ostrakhovitch et al. found that poly(3,4-ethylenedioxythiophene) (PEDOT) : polyethylene glycol (PEG), ITO, and fluorine doped tin oxide (FTO) glass slides can facilitate the neural differentiation of mouse NSE and P19 pluripotent embryonal carcinoma cells and greatly increase the expression of *β*III-tubulin [72]. However, Stewart et al. showed that ES in PPy-containing dopant dodecylbenzenesulfonate (DBS) can predominantly induce the differentiation of NSCs into neurons and less likely into glial cells [65]. It remains unclear whether ES can manipulate the differentiation of stem cells into specific subtypes of neurons, including glutamatergic or dopaminergic neurons.

### 3.2. Effect of Electrical Stimulation through 3D Conductive Material on Stem Cell Neural Differentiation

**Figure 2:**
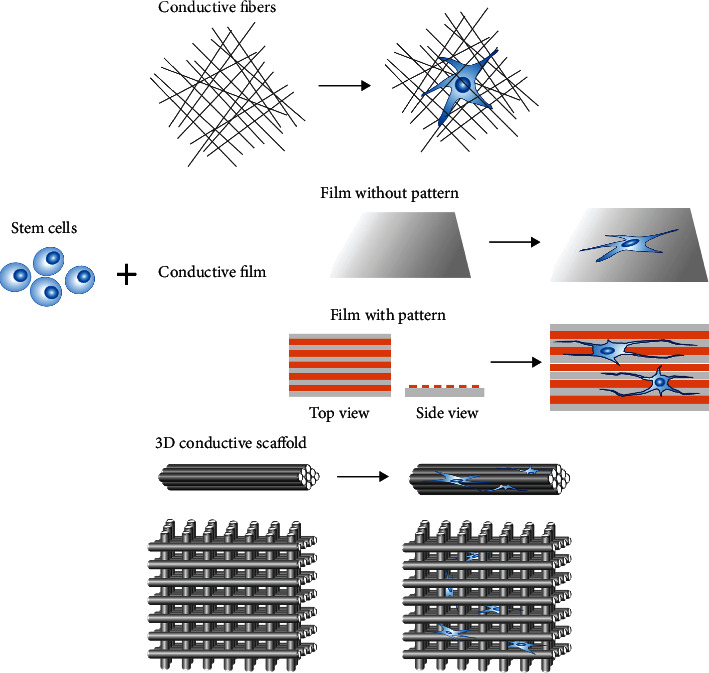
Schematic diagram of structures of conductive materials used for neural tissue engineering.

Compared to 2D cell monolayers, stem cells cultured in a 3D model showed improved cell behavior [73–75]. Numerous materials such as electroconductive hydrogels [76], carbon nanotubes [69, 77], and other nanocomponents [78] have been utilized in developing 3D stem cell neuronal differentiation model [79].  Figure 2 shows the structures of conductive materials used for neural tissue engineering. Heo et al. reported that 3D cultured adipose-derived stem cells formed distinct cell spheres in poly(3,4-ethylenedioxythiophene) : polystyrene sulfonate (PEDOT : PSS) microwells and showed higher neuronal gene expression levels with ES [80]. Rahmani et al. [81] used silk fibroin and reduced GO to generate a 3D conductive nanofibrous scaffold that delivered pulsed current (2 : 115 V/m, 0.1 and 1 : 115 V/m, 100 Hz). Their conductive fibrous scaffold promoted conjunctiva MSCs to differentiate into neural cells by upregulating neural genes, such as MAP2, *β*III-tubulin, and NSE. Carbon nanomaterials, such as graphene nanoplatelets (GNPs) and multiwalled carbon nanotubes (MWCNTs), also demonstrated the ability to enhance cell proliferation and neurite outgrowth and differentiation [82–84].

3D printing is an emerging manufacturing technology with great potential in tissue engineering as it provides a powerful fabrication method for generating accurate and complex patterns and architectures with biochemicals and cells. Particularly, 3D printed platforms are being used for neural regeneration [85–87]. Hydrogels, biodegradable polymers, and novel biomaterials have been used in 3D printing. To date, various 3D printed scaffolds made of different materials have demonstrated their high potential in neural tissue engineering and regeneration [77, 88, 89]. For example, an aqueous dispersion mixture of cellulose nanofibrils (CNF) and single-walled carbon nanotubes (CNT) was used as conductive ink to print guidelines for culturing neural cells (SH-SY5Y) [90]. An amine functionalized MWCNT and polyethylene glycol dipropionate (PEGDA) polymer composite complex was fabricated into a tunable porous neural scaffold that could promote neural stem cell proliferation and neuronal differentiation via a stereolithography 3D printer [77]. Petrella et al. used a 3D printer anchored with a picosecond pulse electric field electrode to print MSCs and NSCs [38]. Their data indicated that 40 kV/cm at 1800 pulses can promote astrocyte specific differentiation but not alter differentiation of MSCs. Aggas et al. also printed 3D hybrid soft conductive hydrogel to support PC12 (a rat pheochromocytoma cell line) attachment [91]. In addition, stem cells and neurites have been shown to grow and extend in the direction of aligned fibers, respectively [92, 93]. Differentiated neural cells have been reported to present higher expression levels of neuronal differentiation markers and better properties than random fibers [94–96]. In summary, 3D printed conductive nanomaterials offer great advantages for stem cell neural differentiation owing to better morphological control, in addition to biochemical cues.

## 4. Potential Mechanism of Electrical Stimulation on Neural Differentiation

**Figure 3:**
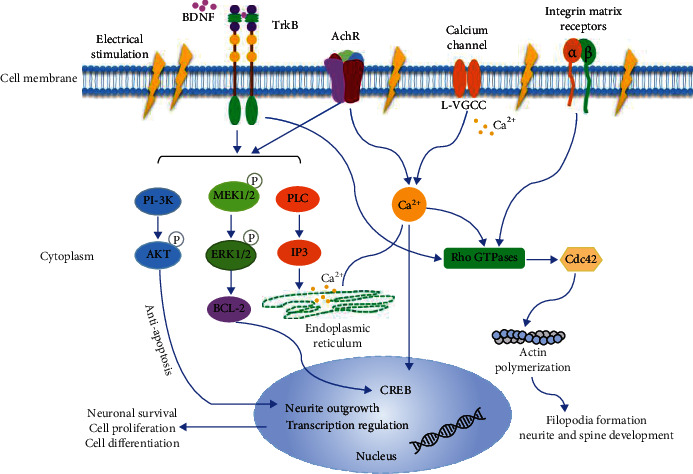
Potential mechanism of electrical stimulation on neural differentiation.

In addition to neurotrophic factors, physical stimulation such as ES can also promote neural differentiation. ES can promote stem cell proliferation [24], migration [2], and neuronal differentiation. It regulates the cell differentiation via a complex mechanism, including changes in the extracellular matrix, cell surface receptor activation, microfilament reorganization, Ca^2+^ dynamics, and many intracellular signaling pathways. Here, we summarize the potential underlying mechanisms (Figure 3).

The mechanism of electrical current guided migration of neurites and cells varies among cell types. Several studies have demonstrated that the PI3K/Akt and mitogen-activated protein kinase (MAPK)/extracellular signal-regulated kinase (ERK) pathways are involved in regulating NSC migration under ES [42, 102–104]. Dong et al. demonstrated that the expression of Ascl1 is required for ES-induced neuronal differentiation of NSCs and that their expression is positively related to the strength of the electric field, regulated by ES; this triggers the activation of the PI3K/Akt pathway [42]. In contrast, Rajnicek et al. found that neuronal growth cones migrating toward the cathode were regulated by cell division cycle 42 (Cdc42), Rac, and Rho and not by the PI3K and MAPK/ERK signaling pathways, which were found in the electric field guidance of nonneuronal cells [105]. Electric field-guided directional migration in iPSCs and neurons depends on Rho-kinase signaling [37]. Feng et al. found that a small DC ES (16 mV/mm) was effective in guiding the migration of human ESC-derived NSCs toward the cathode and that this guidance was not exerted through the Rho/Rho-associated protein kinase or C-X-C chemokine receptor type 4 signaling pathway [35]. Wang et al. have found that the brain-derived neurotrophic factor PI3K/Akt signaling pathway activated by BES can protect against growth factor-deprived NSC apoptosis [55].

Moreover, in an in *vivo* test on rats, ES increased the expression and phosphorylation of ERK1/2 (pERK1/2), and pERK1/2 upregulated the expression of the antiapoptotic protein B-cell lymphoma-2, which finally promoted neuronal cell survival. Furthermore, ES upregulated the expression of p38, which inhibited RhoA-induced neurite outgrowth and neuronal differentiation. These two pathways can lead to the neuronal regeneration and recovery of the electrophysiological function of an injured spinal cord [44]. Chang et al. demonstrated that the combination of nerve growth factor and ES promote neurite outgrowth by increasing the activity of protein kinase C and pERK1/2 [106].

Ca^2+^ is an important signaling ion involved in various biological activities. Studies have shown that Ca^2+^ influx is important for stem cell fate determination. ES can enhance neural growth toward neurotrophic growth factors by increasing cytoplasmic Ca^2+^ and cyclic adenosine monophosphate (cAMP) [107]. Masahisa et al. found that Ca^2+^ contribute to ES and enhance the neuronal differentiation of ESCs [36]. As a whole, the fundamental mechanism of ES-promoting stem cell neural differentiation is quite complicated, and further research is imperative to completely understand and improve the efficiency of neural regeneration. Coupled with newly developed tools, such as single-cell sequencing and gene editing, these technologies may help identify the ES-induced genes that are crucial for regulating stem cell neural differentiation.

## 5. Conclusions

The use of stem cell-derived neural cells is emerging as an effective therapeutic strategy. Stem cells have been used for transplantation to treat nerve diseases with proven safety and efficacy. For example, MSCs have been proven to be safe and effective in treating multiple sclerosis and ischemic stroke [108, 109]. Many factors are associated with the efficacy of stem cell therapy and regenerative medicine in nerve diseases. The most important of them is finding effective methods to induce neural differentiation.

Stem cell differentiation is a complicated process that is regulated by various external and internal factors. ES is likely involved in neurogenesis. Compared to the use of chemically or biologically induced differentiation, ES has the advantage of precisely controlling the stimulation through on/off switching and the selective stimulation region as the cells exposed to ES can be easily selected according to the placement of needle electrodes or conductive materials. In addition, ES can be accurately manipulated in a time-controlled manner through an external power supply. However, there are some limitations of ES in the regulation of the neural differentiation of stem cells. Various types of currents, such as DC, AC, and pulsed current, are used in ES; thus, the effects of ES on stem cell differentiation are diverse, depending on the cell types and ES conditions. As such, directly comparing studies that use different experimental parameters in many aspects is not possible. Importantly, the timing of ES is an essential factor which can strongly influence stem cell differentiation. Nevertheless, current platforms preclude high-throughput screening to simultaneously study the complicated parameters. In addition, although ES is generally effective, it is not as potent as growth factors. This problem can be solved by combining electrical and biochemical stimulation which can potentially promote the differentiation of stem cells in a more robust and controlled manner. Many studies have proved that combined therapy is a strong rational approach for tissue engineering and nerve disease treatment [110–113]. With the development of materials, micropatterned conductive materials can not only provide ES but also guide cell and neurites orientation through topographies. Some conductive materials, especially nanomaterials, can generate complexed 3D structures to further facilitate scaffold-based cellular transplants. Biomaterial 3D scaffold is one of the most promising approaches for *in vivo* applications, as it not only can provide a biophysical microenvironment but is also easily compatible with various stimulation cues.

At present, except for orthodox treatment, ES has been considered as a useful noninvasive, interventional method in the clinic. Regardless of the types of waveform of the ES, their effects on neural disease in animal models and human patients have been demonstrated. However, there are many factors that can impact the efficiency of ES-based therapy, such as the source of stem cells, parameters of electric, onset timing and duration of ES, and the stimulation interface materials. A fundamental understanding of the most crucial driving mechanism underlying neural differentiation upon ES will greatly improve the experimental reproducibility and clinical translation. We believe that the combination of new conductive materials and stem cells will contribute to the application of stem cell-based therapy for nerve diseases treatment.

## Figures and Tables

**Table 1:** iPSCs and ESCs used in clinical trials for the treatment of nerve diseases.

Cell type/goal	Source	Disease	Phase	Trail number
Oligodendrocyte progenitor cell	Human brain	Demyelinating diseases	Unknown	NCT00283023
Human ESC-derived neural precursor cells	Human embryonic stem cells	Parkinson's disease	Phase 2	NCT03119636
Development of iPSCs	Somatic cells of patients with neurological diseases	Neurodegenerative disorders	Recruiting	NCT00874783
Generate disease-specific iPSC lines	Neuro-degenerative disease patients	Neuro-degenerative disease	Recruiting	NCT03322306
Establishing of neuronal-like cells from iPSCs	PBMCs	Peripheral nervous system diseases	Withdrawn (lack of funding)	NCT02492360
Neurons and glia derived from iPSCs	Patients with genetic mutations responsible for neurological and neurodegenerative diseases	Neurodegenerative diseases	Not yet recruiting	NCT03682458
Develop human iPSCs	An existing collection of human somatic cells	Amyotrophic lateral sclerosis	Recruiting	NCT00801333
Establishment of human cellular disease models from iPSCs	Patient-derived fibroblasts	Wilson disease	Recruiting	NCT03867526
Neuronal distinction of iPSC	Human fibroblast with MYT1L mutation	Mental retardation	Completed	NCT02980302
Neuronal progenitors derived from iPSC	Blood sample	Rare intellectual disabilities	Recruiting	NCT03635294
Neural cells derived from iPSC	Patients' skin	Niemann-pick diseases	Recruiting	NCT03883750
Establish an iPSC bank	Patients with NF1 mutations	Tumors in the central nervous system	Suspended	NCT03332030
Derivation of iPSC	Human somatic cells from existing collections	Amyotrophic lateral sclerosis	Recruiting	NCT00801333
Creation of a large repository of iPSC	Blood and spinal fluid (optional)	Amyotrophic lateral sclerosis	Completed	NCT02574390
Creation of a Bank of Fibroblast from iPSC	Skin biopsy	Amyotrophic lateral sclerosis	Completed	NCT01639391
Development of iPSC	Patients' fibroblast	Neurodegenerative disorders	Recruiting	NCT00874783

**Table 2:** Direct current stimulation used in stem cell neural differentiation.

ES type	Cell type	Conductive material	Stimulation parameters	ES effect	Reference
DC	NSCs	Two parallel Ag/AgCl wires	115 V/m, 2 hours/day for two days	Enhanced undifferentiated cell mobility and directional migration, and differentiation towards *β*III-tubulin+ neurons	Zhao H et al. [39]
DC	NSCs	Platinum electrodes	0.53 or 1.83 V/m, 10 min/days for 2 days	Increased neurites length, and *β*III-tubulin, NeuN gene expression and in intracellular Ca^2+^	Kobelt LJ et al. [40]
DC	MSCs	Two parallel 316 L stainless steel electrodes, PANI films	1 mV-2 V, 10 min/day, 3 days	Enhanced filopodial elongation, increased nestin and *β*III-tubulin gene expression	Thrivikraman G et al. [41]
DC	NSCs	Poly-D-lysin/lamini-coated electrotactic chambers	150 mV/mm, 7, 14 days	Enhanced neural differentiation (Ascl1, *β*III-tubulin, MAP2 gene expression)	Dong ZY et al. [42]
DC	Coculture of C2C12 with hMSCs	Two parallel electrodes	8 mV/mm, 20 h/day, 8 days	Increased neural markers (SOX2, nestin, *β*III-tubulin) gene level and intracellular Ca^2+^ activity	Naskar S et al. [43]

**Table 3:** Alternating current stimulation used in stem cell neural differentiation.

ES type	Cell type	Conductive material	Stimulation parameters	ES effect	Reference
AC	NSCs	Ag/AgCl electrodes	46 mV/mm, 0.5 Hz	AC ES showed no differences in alignment or differentiation	Ariza CA et al. [51]
AC	NSCs	Nickel-coated wire electrodes, alginate beads	0.1–10 Hz, 2, 4, 16 V/m, 7, 14, 21 days	Increased ratio of neurons to astrocytesneural and stem cell viability under lower frequence	Matos MA et al. [52]
AC	Porcine NSCs	Two gold contact pads connected to 25 electrode pairs	1–50 Hz, 0.001 kV/cm	Delayed differentiation into astrocytes	Lim JH et al. [53]

**Table 4:** Pulsed current stimulation used in stem cell neural differentiation.

ES type	Cell type	Conductive material	Stimulation parameters	ES effect	Reference
Pulsed current	ESCs	4-mm gap cuvette	0, 5, 10, and 20 V, 5 pulses (950 ms interpulse interval)	Increased differentiate into various types of neurons in vivo	Yamada M et al. [36]
Pulsed current electric field	NSCs and MSCs	1 cm long parallel electrodes	20 and 40 kV/cm, 24 h, 503 ps, amplitude of 1016 V/m,	Upregulation of NSCs astrocyte specific differentiation	Petrella RA et al. [38]
Biphasic electrical stimulation (BES)	Olfactory bulb NSCs	Fluorine-doped tin oxide glass plates	25 mV/mm and 50 mV/mm, 8 ms pulses (20% duty cycle), 12 h	Improving cell survival and preventing cell apoptosis	Wang L et al. [55]
BES	Fetal NSCs	ITO glasses electrodes	100 Hz,4, 8, 16 and 32 mA/cm^2^ with 50 and 200 ms pulses, 4 or 7 days	Promote both the proliferation and neuronal differentiation	Chang KA et al. [56]
Pulsed electrical stimulation	Neuro-spheres	ITO electrodes	5 V, 30 Hz	Enhanced *β*III-tubulin and calcium influxes	Tandon N et al. [57]
Pulsed current	Human neural crest stem cell	Au electrodes placed in a top bottom of 96 well plate	2 or 20 Hz, 100 *μ*s, 200 mV/mm, 24 h	Enhanced nerve regeneration, increased Schwann cell differentiation	Du J et al. [58]
Pulsed current	Mouse NSC	Ag/AgCl electrodes	300 mV/mm, 100 Hz, 50% duty cycle, 48 h	Induced NSCs differentiation into neurons, astrocytes, and oligodendrocytes simultaneously	Chang HF et al. [59]
Pulsed current	NSCs	PLGA/GO conductive composite membrane	100 mV, 20, 100, and 500 Hz, 1 h/day, 3 days	Promote cell migration, adhesion and proliferation rates; promote neurite elongation and neuron differentiation, inhibited astrocytes differentiation	Fu C et al. [60]
Pulsed electric simulation a self-powered electrical simulation system	MSC	Reduced GO-PEDOT hybrid microfiber	300 V, 30 *μ*A, 21 days	Increased *β*III-tubulin and GFAP gene expression	Guo W et al. [61]

**Table 5:** Electrical stimulation through conductive materials for stem cell neural differentiation.

Conductive material	ES type	Cell type	Dimension	Stimulation parameters	ES effect	Reference
Crosslinked PEDOT : PSS films	Pulsed electrical stimulation	NSCs	2D	100 Hz, 1 V, 10 ms, 24 h first 4 days, 12 h/day for 8 days,	Increased Tuj1+ neuron ratio and neurites length	Pires F et al. [27]
PLGA/GO conductive composite membrane	Pulsed current	NSCs	2D	100 mV, 20, 100, and 500 Hz, 1 h/day, 3 days	Promote cell migration, adhesion and proliferation rates; promote neurite elongation and neuron differentiation, inhibited astrocytes differentiation	Fu C et al. [60]
Ti-coated nanopatterned substrate	Pulsed electrical stimulation	NSCs	2D	3 *μ*A, 25 V, 1 Hz, 30 min, twice a day	Upregulated expression of the neuronal markers Tuj1 and NeuN	Yang K et al. [63]
PPy containing the anionic DBS	Pulsed current	NSCs	2D	±0.25 mA/cm^2^, 100 ms pulses, 250 Hz	Predominantly induced NSCs differentiation into neurons, less glial	Stewart E. et al. [65]
p(HEMA-co-HMMA-co-PEGMA) hydrogels	AC	PC12	2D	N/A	Supported cell attachment, but not the differentiation	Aggas JR et al. [91]
PPy electroplated onto ITO slides	Pulsed current	NPCs derived from the H9 human ESCs	2D	+1 V to −1 V, 1 kHz for 1 h	Enhanced stroke recovery after transplanted into stroke injured rats	George, PM et al. [97]
PANI/PG	DC	NSCs	2D	1.5 V for 15, 30, and 60 min	Enhanced the cell proliferation and neurite outgrowth	Ghasemi-Mobarakeh L et al. [98]
GNPs and MWCNTs	DC	HT-22	2D	4.9335*E*−6 S/m (GNPs); 1.89875*E*−5 S/m (MWCNTs), days 1, 3, and 5	Reinforced cell proliferation and induced elongated morphology	Gupta P et al. [82]
Reduced GO-PEDOT hybrid microfiber	Pulsed electric simulation a self-powered electrical simulation system	MSCs	3D	250 V, 30 *μ*A, 21 days	Induced high Tuj1 and GFAP gene expression	Guo W et al. [61]
PEGDA incorporated carbon nanotubes	Biphasic pulse	NSCs	3D	100, 500, 1000 *μ*A, 100 Hz	Promoted cell proliferation and oligodendroglial differentiation (Tuj1, GFAP expression)	Lee SJ et al. [77]
BC/PEDOT nanofibers	Monophasic anodic pulses	PC12	3D	1–100 ms	Increased PC12 action potentials	Chen C et al. [78]
CNF/CNT ink	DC	SH-SY5Y	3D	3.8 × 10^−1^ S cm^−1^, 10 days	Direct and enhance neural cell development	Kuzmenko V et al. [90]
3D graphene scaffold	Pulsed current	Patient-iPSC derived neural progenitcells	3D	10 *μ*A, 1 Hz, 30 min/day for 3 days	Increased cell maturation (Tuj1 and MAP2 expression)	Nguyen AT et al. [99]
Polypyrrole-coated poly lactic acid fibrous	Biphasic potential	NSCs	3D	100 mV, 50 Hz for 3 days	Enhanced cell migration and neurite outgrowth	Sudwilai Thitima et al. [100]
Silk scaffold	Pulsed current	Primary neuron	3D	160 mV, 0.5 Hz–2 kHz, 24 h	Induced axon alignment and growth	Tang-Schomer MD et al. [101]

BC: bacterial cellulose; PEDOT: poly(3,4-ethylenedioxythiophene); PPy: polypyrrole; PANI: polyaniline; PG: poly (ɛ-caprolactone)/gelatin; GO: graphene oxide; PLGA: poly (L-lactic-co-glycolic acid); Ti: titanium; ITO: indium tin oxide; NPCs: neural progenitor cells; DBS: dopant dodecylbenzenesulfonate.

## Data Availability

All data included in this study appear in the submitted article.
